# Seasonal differences in cardiac prognosis in incident hemodialysis patients: a finding from Japanese multicenter dialysis cohort study

**DOI:** 10.1007/s10157-025-02768-8

**Published:** 2025-10-29

**Authors:** Yuki Fujishima, Daijo Inaguma, Shimon Kurasawa, Masaki Okazaki, Takahiro Imaizumi, Shoichi Maruyama

**Affiliations:** 1 Department of Nephrology, Nagoya University Graduate School of Medicine, 65 Tsurumai-Cho, Showa-Ku, Nagoya, Aichi 466-8550 Japan; 2 Department of Internal Medicine, Fujita Health University Bantane Hospital, 3-6-10 Otobashi, Nakagawa-Ku, Nagoya, Aichi 454-8509 Japan; 3 Department of Nephrology, Kariya Toyota General Hospital, 5-15 Sumiyoshi-Cho, Kariya, Aichi 448-8505 Japan; 4 Department of Clinical Research Education, Nagoya University Graduate School of Medicine, 65 Tsurumai-Cho, Showa-Ku, Nagoya, Aichi 466-8550 Japan; 5 Department of Advanced Medicine, Nagoya University Hospital, 65 Tsurumai-Cho, Showa-Ku, Nagoya, Aichi 466-8550 Japan

**Keywords:** Seasonal difference, Hemodialysis, Cardiac disease

## Abstract

**Background:**

The initiation of hemodialysis exhibits winter-peak seasonal variations, possibly associated with increased cardiac events during winter. The season of cardiac disease onset affects prognosis; however, the relationship between the season of hemodialysis initiation and subsequent cardiac outcomes remains unclear. We aimed to evaluate this association to determine whether the season of hemodialysis initiation could influence subsequent cardiac events.

**Methods:**

We used data from a Japanese multicenter prospective dialysis initiation cohort. We divided the patients into four groups based on the season of hemodialysis initiation: Spring, Summer, Autumn, and Winter. The outcome was 3-year cardiac events defined as a composite of ischemic heart disease, heart failure, and sudden death. Considering the competing risks, we compared the incidence of subsequent cardiac events with the hemodialysis initiation season.

**Results:**

Among the 1396 eligible patients, hemodialysis was initiated in 402 (29%), 346 (25%), 270 (19%), and 378 (27%) patients in Spring, Summer, Autumn, and Winter, respectively. Total fluid removal, heart failure symptoms, and fluid overload during the first hemodialysis session were more frequent in Autumn and Winter. During the 3-year follow-up, 264 patients (19%) developed cardiac events. Autumn was associated with a higher risk of developing cardiac events than Summer. Compared with Summer, the adjusted subdistribution hazard ratios (95% confidence intervals) were 1.40 (0.97–2.02) in Spring, 1.50 (1.02–2.21) in Autumn, and 1.15 (0.80–1.67) in Winter.

**Conclusion:**

Hemodialysis initiation in autumn may be a potential indicator of subsequent cardiac events. Further studies are required to elucidate the underlying pathophysiological mechanisms.

## Introduction

Studies have reported winter-peak and summer-nadir seasonal variations in hemodialysis initiation from the United States and Japan [1–6]. A Japanese single-center study reported that among patients aged ≥ 75 years, fluid overload was the most common cause of emergency hemodialysis initiation in winter (45%) [5]. Notably, emergent hemodialysis initiation due to fluid overload showed seasonal variation, peaking in winter, whereas cases caused by uremia or abnormal laboratory results showed no seasonal difference. These findings suggest that cardiac disease, whether clinical or subclinical, may play a role in hemodialysis initiation throughout the year, including the increased initiation observed during winter. However, the seasonal distribution of hemodialysis initiation does not directly mirror overall cardiac risk, as different seasons may disproportionately affect patient populations with distinct cardiac vulnerability profiles. Acute myocardial infarction and heart failure occurring during winter or cold seasons are reported to have the highest in-hospital mortality and lowest cumulative survival that is free from major adverse cardiac events compared with other seasons [7, 8]. Nevertheless, the relationship between the season of hemodialysis initiation and subsequent cardiac disease remains unclear.

Patients undergoing dialysis have high morbidity and mortality from heart failure, coronary artery disease, and sudden cardiac death [9–15]. Recently, patients frequently have traditional cardiovascular risk factors like hypertension, diabetes mellitus (DM), and left ventricular hypertrophy (LVH). Simultaneously, they have non-traditional risk factors like malnutrition and chronic kidney disease–mineral bone disorder, some of which are specific to chronic kidney disease [16, 17]. Despite ongoing efforts to manage these risk factors, cardiac diseases, including ischemic heart disease, heart failure, and sudden death, remain the leading causes of death in chronic hemodialysis patients, causing approximately 35% of all deaths in the United States [18]. In Japan, heart failure and myocardial infarction account for approximately 25% of the deaths among patients undergoing dialysis [19].

We examined the association between hemodialysis initiation season and subsequent cardiac disease development. This investigation could help identify patients at a higher risk of future cardiac events, enabling the implementation of optimal preventive strategies earlier in their lives on hemodialysis.

## Materials and methods

### Study design, setting, and population

We used the database of the Aichi Cohort Study of Prognosis in Patients Newly Initiated into Dialysis (AICOPP), a prospective, multicenter cohort study consisting of 1520 adult patients, newly initiated on dialysis between October 2011 and September 2013 in Aichi Prefecture (Japan) [20]. Patients who died during hospitalization for dialysis initiation or were treated with renal replacement therapy due to acute kidney failure in the original cohort were excluded. In this study, we included all participants of the AICOPP who had initiated hemodialysis. We excluded participants with a history of peritoneal dialysis and those with a missing transition date to peritoneal dialysis. We reported the frequency of hemodialysis initiation as the average number per day because the number of days included in each month or season varied. The patients were divided into four groups based on the month of hemodialysis initiation following the definition provided by the Japan Meteorological Agency [21]: Spring (March–April–May), Summer (June–July–August), Autumn (September–October–November), and Winter (December–January–February).

### Collected data in AICOPP and variable definitions

Briefly, the AICOPP obtained data on age, sex, comorbidities, medications, symptoms, vital signs, laboratory and imaging findings at the initiation of dialysis, vascular access, and modality of the first dialysis. Body mass index (BMI) was calculated by dividing a person’s weight by the square of their height. The left ventricular ejection fraction (LVEF) was calculated using the following formula based on echocardiographic findings: LVEF (%) = (left ventricular diastolic volume–left ventricular systolic volume)/left ventricular diastolic volume × 100. Data on disease onset and deaths among the AICOPP participants were collected by contacting maintenance dialysis clinics every 6 months between March 2014 and September 2016. Heart failure cases were defined as a history of hospitalization for heart failure. The renin-angiotensin system inhibitors include angiotensin-converting enzyme inhibitors and angiotensin II receptor blockers. Diuretics included loop diuretics, thiazides, and spironolactones. Total fluid removal was calculated as the difference between the pre- and post-dialysis weights.

### Outcome definition and follow-up period

The primary outcome of this study was cardiac events, defined as a composite of ischemic heart disease, heart failure, and sudden death. Ischemic heart disease was defined as the incidence of or death from coronary artery disease, angina pectoris (except for vasospastic angina), percutaneous coronary intervention, or coronary artery bypass graft surgery; heart failure as hospitalization or death due to heart failure; and sudden death as sudden death or death from a fatal arrhythmia. The competing risk was non-cardiac death, defined as death from causes other than cardiac or from unknown causes. Patients were followed up from the date of hemodialysis initiation until the incidence of the first cardiac event or death for up to 3 years, which was the minimum follow-up period in AICOPP. Patients lost to follow-up were censored at the last confirmed contact. We also censored patients who discontinued dialysis, switched to peritoneal dialysis or kidney transplantation, or died during follow-up.

### Statistical analysis

We analyzed continuous variables that were normally distributed (mean and standard deviation) using ANOVA. Kruskal–Wallis test was used for continuous variables with non-normal distributions (median and interquartile range). The chi-squared test was used to compare variables by group for binary or categorical variables. We used a competing risk model to evaluate the influence of the hemodialysis initiation season on the incidence of cardiac events, accounting for the effects of competing risks. We presented the number and rates of cardiac events, their components, and non-cardiac deaths by group. We conducted a cumulative incidence function and Gray’s test. We conducted a competing risk multivariate regression analysis using the Fine-Gray model. Analyses were conducted using unadjusted and two multivariate-adjusted models adjusted for the following covariates: Model 1, patient characteristics (age, sex, comorbid DM, history of heart failure admission, and history of coronary artery disease); Model 2, Model 1 plus laboratory findings (serum hemoglobin, albumin, creatinine, low-density lipoprotein cholesterol (LDL-C), corrected calcium, phosphorus, log-intact parathyroid hormone, and log-C-reactive protein levels) and situation (temporary catheter use) at hemodialysis initiation. The following variables had > 3% missing values: LVEF (18%), intact parathyroid hormone (13%), C-reactive protein (CRP) (5.9%), LDL-C (14%), and hemoglobin A1c (24%). The remaining variables had missing values of < 3%. We conducted multiple imputations using chained equations to impute all missing data and combined the results of the competing risk regression from the 20 imputed datasets using Rubin’s rule. We also performed the subgroup analysis with the imputed dataset by age (≥ 75 and < 75 years), sex, comorbid DM, history of admission due to heart failure, and history of coronary artery disease. All statistical analyses were conducted using Stata/SE 17.0 (StataCorp, College Station, TX, USA), except for Gray’s test conducted using EZR (Saitama Medical Center, Jichi Medical University, Saitama, Japan), which is a graphical user interface for R (The R Foundation for Statistical Computing, Vienna, Austria). Statistical significance was set at *p* < 0.05.

## Results

**Fig. 1:**
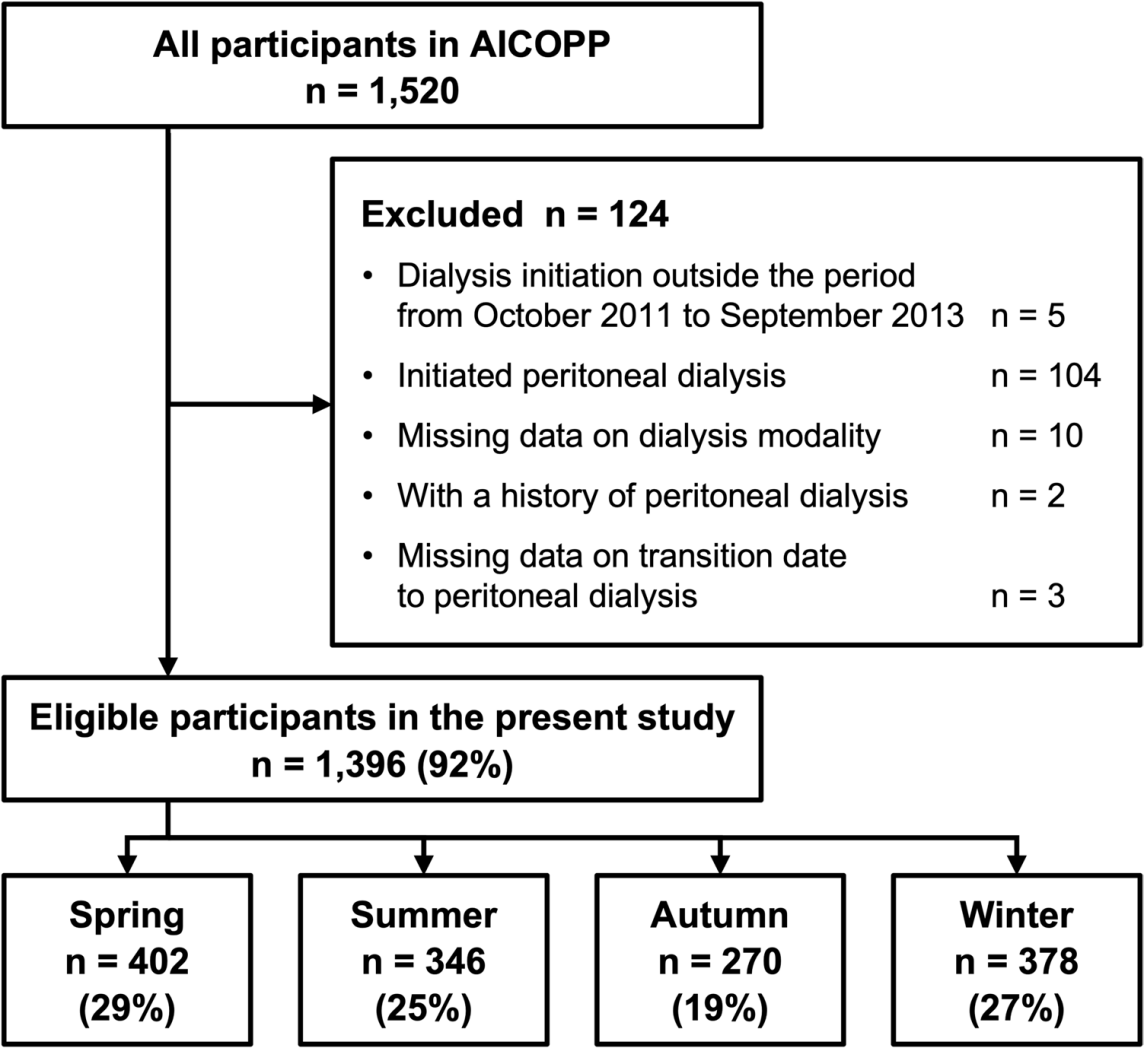
Flow chart of patient selection and grouping

**Fig. 2:**
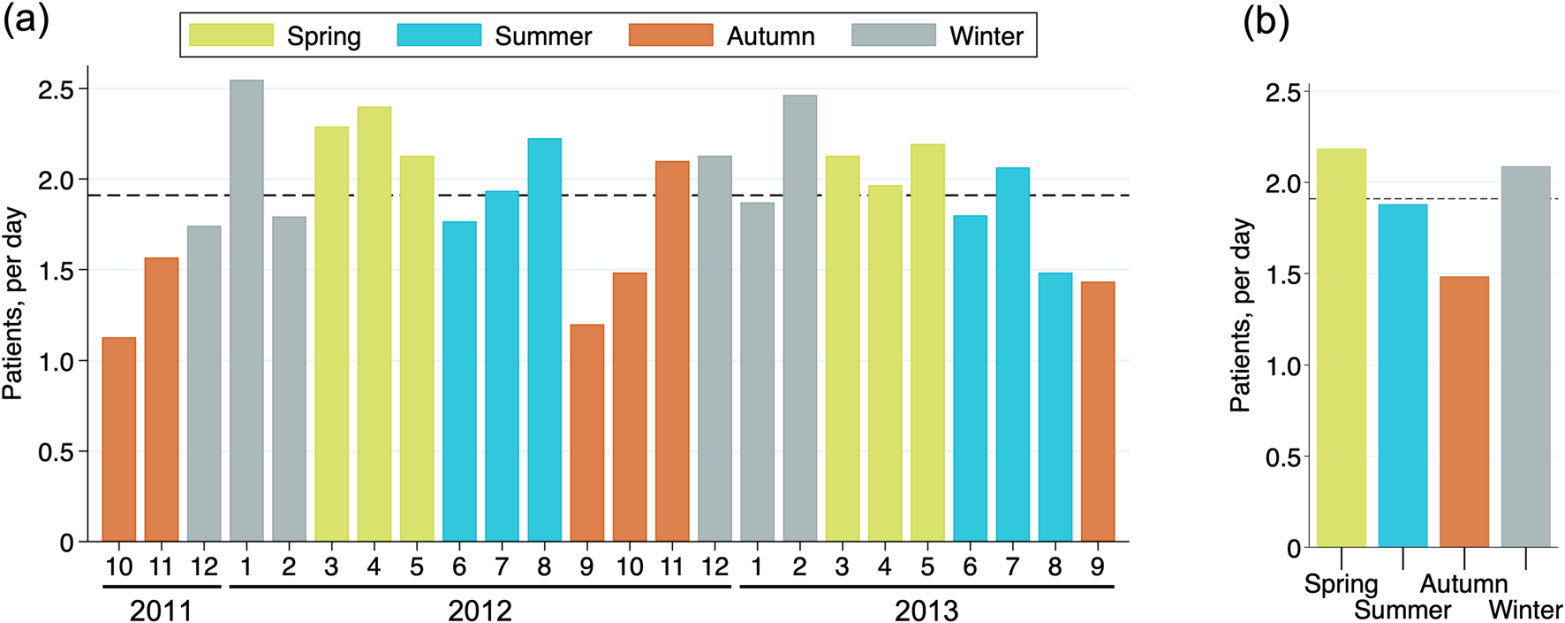
Number of patients starting hemodialysis per day by month **a** and season **b**. The dashed line shows the overall average number of hemodialysis initiations: 1.91 patients per day

Overall, 1,396 (92%) out of 1,520 AICOPP participants were eligible for inclusion. We identified 402 (29%), 346 (25%), 270 (19%), and 378 (27%) patients in Spring, Summer, Autumn, and Winter, respectively (Fig. 1). Hemodialysis initiation was most common between December and July and least common between September and October. Seasonally, initiation peaked in spring and was lowest in autumn (Fig. 2).

### Baseline characteristics at hemodialysis initiation and features of the first hemodialysis session

Several baseline characteristics differed significantly between the groups (Table 1). BMI, CRP level, total fluid removal at first hemodialysis, heart failure symptoms, and fluid overload were higher in Autumn and Winter than in Spring and Summer. Primary kidney disease distributions showed apparent differences between the groups, though not statistically significant: diabetic kidney disease was proportionally highest in Autumn and lowest in Summer, renal sclerosis was least represented in Summer, and chronic glomerulonephritis was most represented in Summer. Patients in the Autumn group had higher proportions of males, as well as DM, coronary artery disease history, LDL-C level, and predialysis systolic blood pressure, though these differences did not reach significance. LVEF was similar across the groups. Use of renin-angiotensin system inhibitors was the lowest in Autumn and the second highest in Summer. Similarly, diuretic use was less frequent in Autumn than in Winter or Spring. Both differences were non-significant.

**Table 1:** Baseline characteristics by season of hemodialysis initiation

	Spring (n = 402)	Summer (n = 346)	Autumn (n = 270)	Winter (n = 378)
Age (years)	69 ± 13	68 ± 13	68 ± 12	67 ± 14
Male sex (%)	65	68	72	66
Cause of ESKD (%)				
Chronic glomerulonephritis	12	16	10	14
Diabetic kidney disease	44	42	49	44
Renal sclerosis	27	22	26	27
Others	17	21	15	15
Body mass index (kg/m^2^)*	23.2 ± 3.8	23.1 ± 3.9	23.8 ± 4.6	24.1 ± 5.1
Comorbid or history of disease and medication				
Diabetes mellitus (%)	53	53	60	54
Coronary artery disease (%)	16	15	20	18
Heart failure (%)	18	19	21	26
Stroke (%)	15	15	18	19
Renin-angiotensin system inhibitors (%)	63	62	56	58
Diuretics (%)	73	67	68	73
Statin (%)	38	40	43	41
Laboratory and imaging findings				
Hemoglobin (g/dL)	9.3 ± 1.6	9.4 ± 1.6	9.4 ± 1.6	9.2 ± 1.5
Serum albumin (g/dL)	3.2 ± 0.5	3.2 ± 0.6	3.1 ± 0.6	3.2 ± 0.6
Creatinine (mg/dL)	9.0 ± 3.2	9.0 ± 3.2	9.0 ± 3.6	9.0 ± 3.2
Potassium (mEq/L) *	4.5 ± 0.8	4.4 ± 0.8	4.6 ± 0.9	4.7 ± 0.9
Corrected calcium (mg/dL)	8.6 ± 1.1	8.7 ± 1.0	8.6 ± 1.1	8.5 ± 1.2
Phosphorus (mg/dL)	6.5 ± 2.0	6.3 ± 1.8	6.2 ± 1.9	6.4 ± 2.0
Intact parathyroid hormone (pg/mL)	311 (202–445)	278 (176–447)	275 (187–416)	293 (190–428)
C-reactive protein (mg/dL) *	0.3 (0.1–1.2)	0.2 (0.1–1.2)	0.4 (0.1–1.8)	0.4 (0.2–1.8)
Hemoglobin A1c (%)	5.6 ± 0.9	5.7 ± 1.0	5.6 ± 0.9	5.6 ± 0.9
Low-density lipoprotein cholesterol (mg/dL)	92 ± 34	88 ± 34	94 ± 38	88 ± 33
Left ventricular ejection fraction (%)	63 (56–69)	62 (56–69)	62 (54–69)	63 (53–68)
Feature of the first hemodialysis session				
Temporary catheter use (%)	22	20	24	22
Total fluid removal (mL/session) *	700 (100–1300)	600 (100–1100)	1000 (400–1700)	900 (300–1700)
Pre-dialysis systolic blood pressure (mmHg)	151 ± 25	152 ± 27	153 ± 28	152 ± 26
Heart failure symptoms (%)*	31	25	34	37
Fluid overload (%)*	53	44	59	56

Data are presented as mean ± standard deviation or median (interquartile range) for continuous measures and as percentages for categorical measures

*ESKD* end-stage kidney disease

*p < 0.05

### 3-year cardiac event outcomes

**Fig. 3:**
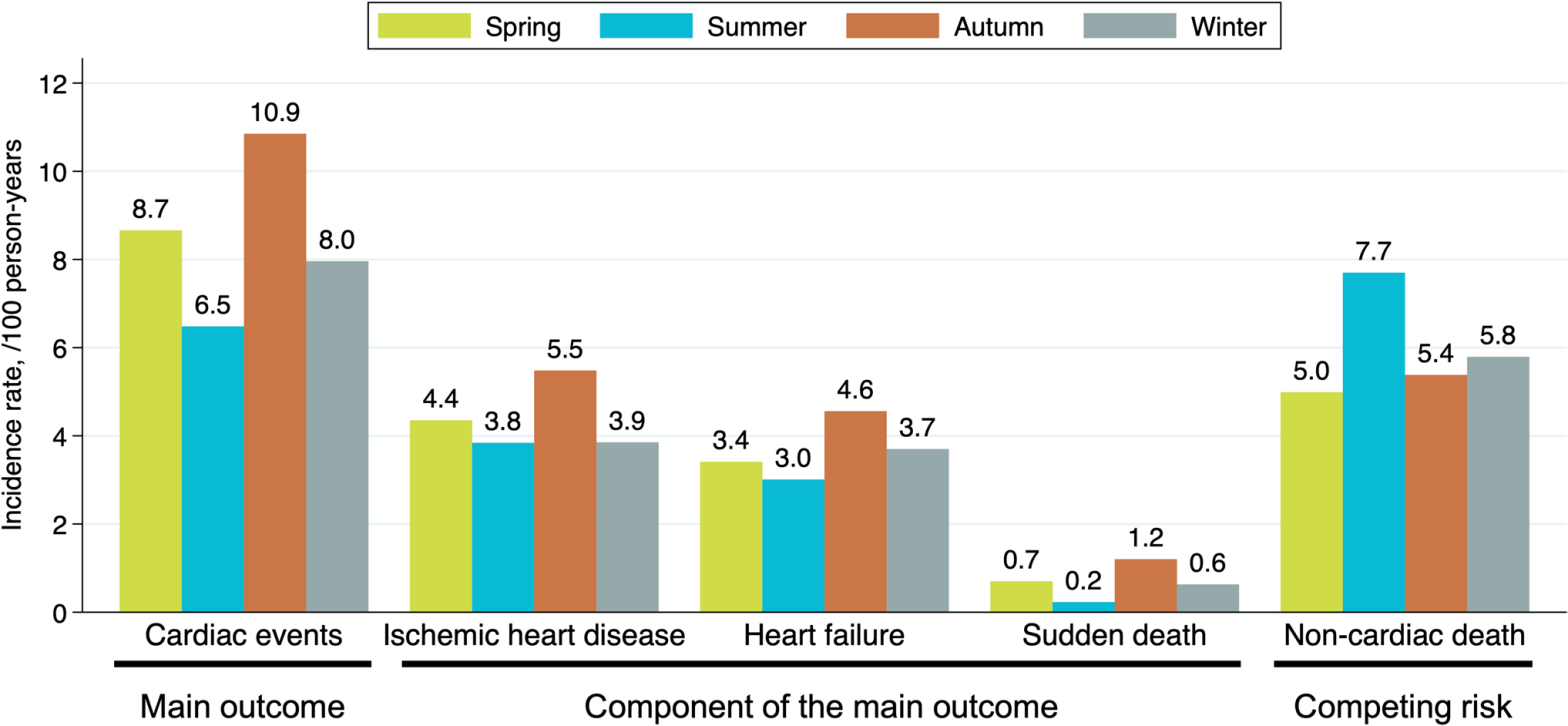
Incidence rates of cardiac events, each component of cardiac events, and non-cardiac death by hemodialysis initiation season. Analysis of the incidence rate of the components was performed independently for each component, with some patients experiencing more than one component of the cardiac events

**Fig. 4:**
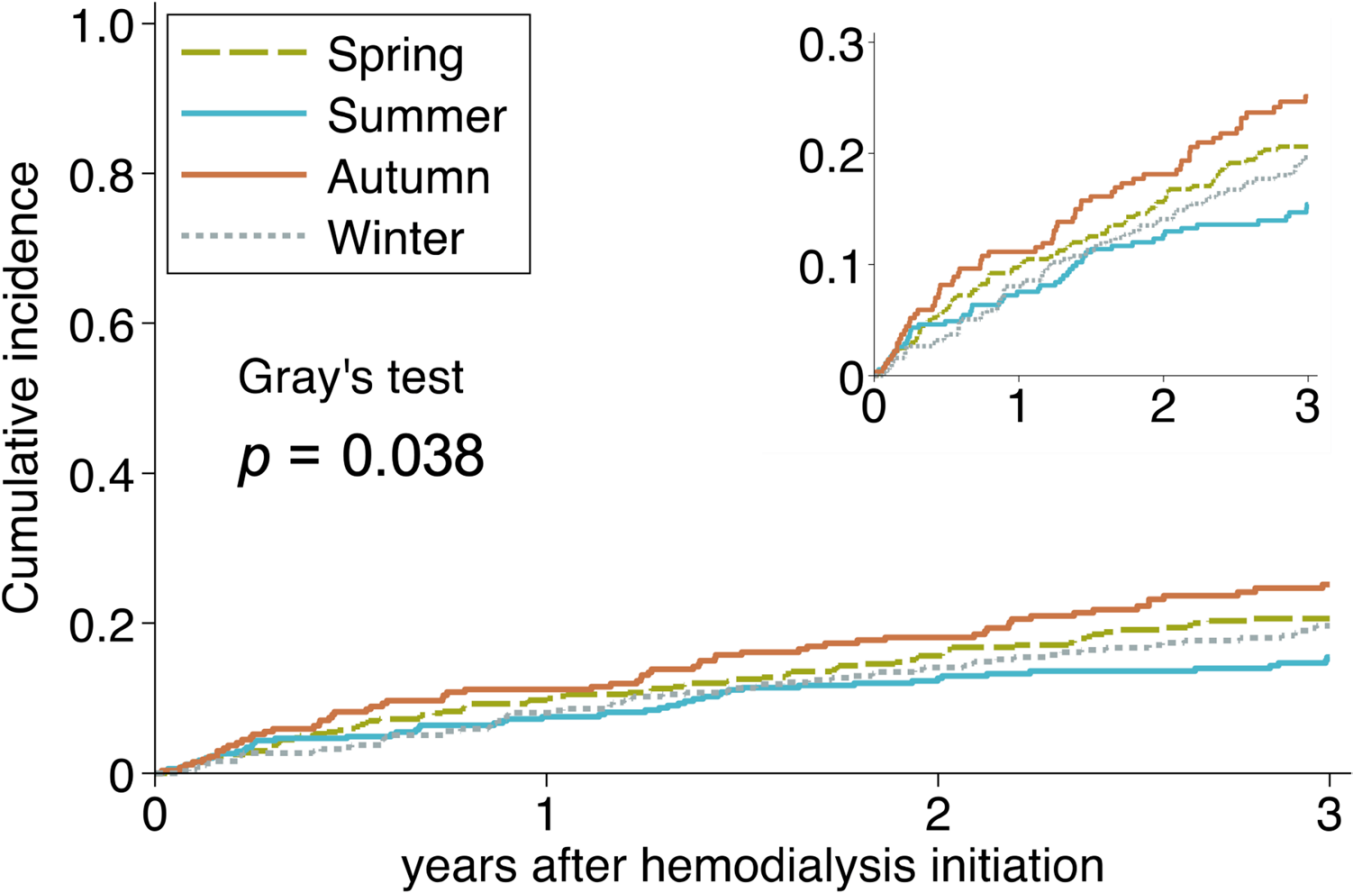
Cumulative incidence function of cardiac events by hemodialysis initiation season,

**Fig. 5:**
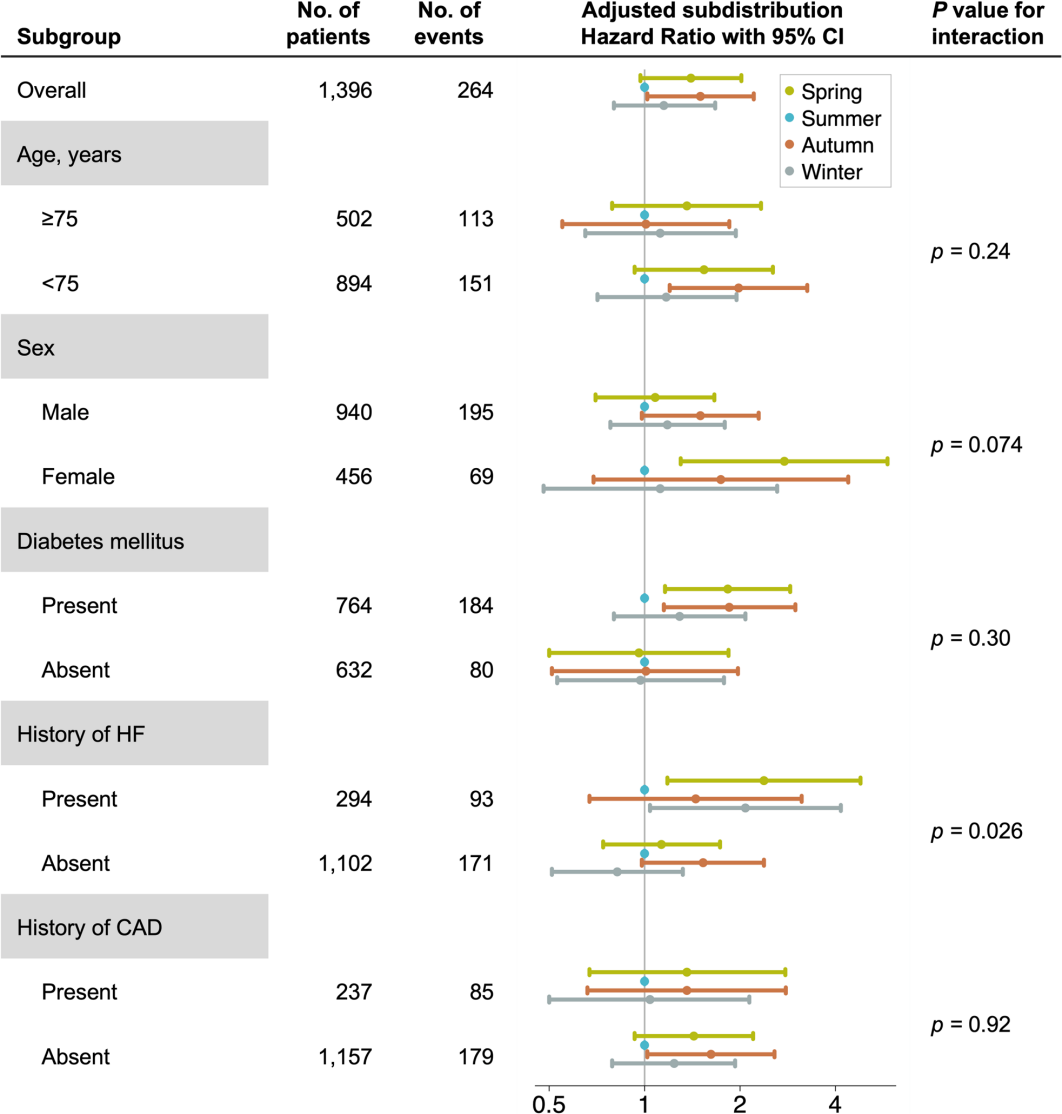
Competing risk regression adjusted subdistribution hazard ratio with 95% Confidence Interval by hemodialysis initiation season in subgroups, Subdistribution hazard ratios were adjusted for patient characteristics (age, sex, comorbid diabetes mellitus, history of heart failure admission, and history of coronary artery disease), laboratory findings at hemodialysis initiation (serum hemoglobin, albumin, creatinine, low-density lipoprotein cholesterol, corrected calcium, phosphorus, log-intact parathyroid hormone levels, and log-C-reactive protein levels), and situation at hemodialysis initiation (temporary catheter use). *HF* heart failure, *CAD* coronary artery disease

During a 3-year follow-up period, 264 (19%) patients experienced cardiac events, and 192 (14%) died from non-cardiac causes without experiencing cardiac events. We censored 234 (17%) patients: 204 (15%) were lost to follow-up, 23 (1.6%) received kidney transplantation, seven (0.5%) discontinued dialysis, and no patients transitioned to peritoneal dialysis. The incidence rates of cardiac events were highest in Autumn and lowest in Summer. Each component of cardiac events showed a similar trend, while non-cardiac death was more frequent in Summer (Table 2 and Fig. 3). The cumulative incidence of cardiac events was higher in the Autumn, Spring, Winter, and Summer groups (*p* = 0.038; Fig. 4). After adjusting for patient characteristics, laboratory findings, and situations at hemodialysis initiation, Autumn was linked to significantly higher risk of developing cardiac events than Summer (adjusted subdistribution hazard ratio, 1.50; 95% confidence interval, 1.02–2.21; Table 3). Only the subgroup analysis based on a history of heart failure showed a statistically significant interaction (Fig. 5).

**Table 2:** Cardiac events, each component of cardiac events, and non-cardiac death by season of hemodialysis initiation

	Spring (n = 402)	Summer (n = 346)	Autumn (n = 270)	Winter (n = 378)
Cardiac events, n (%)	79 (20%)	51 (15%)	64 (24%)	70 (19%)
Components of cardiac events				
Ischemic heart disease, n (%)	41 (10%)	31 (9.0%)	34 (13%)	35 (9.3%)
Heart failure, n (%)	33 (8.2%)	25 (7.2%)	29 (11%)	34 (9.0%)
Sudden death, n (%)	7 (1.7%)	2 (0.6%)	8 (3.0%)	6 (1.6%)
Non-cardiac death, n (%)	50 (12%)	66 (19%)	36 (13%)	55 (15%)

Each component’s total count was higher than that of cardiac events because some patients experienced more than one component

**Table 3:** Subdistribution hazard ratios for cardiac events by season of hemodialysis initiation

	Unadjusted	Model 1^a^	Model 2^b^
Spring	1.37 (0.96–1.95)	1.38 (0.96–1.97)	1.40 (0.97–2.02)
Summer	1.00 (reference)	1.00 (reference)	1.00 (reference)
Autumn	1.70 (1.18–2.47)	1.54 (1.06–2.24)	1.50 (1.02–2.21)
Winter	1.27 (0.88–1.82)	1.17 (0.81–1.68)	1.15 (0.80–1.67)

^a^Model 1 was adjusted for patient characteristics, including age, sex, comorbid diabetes mellitus, history of heart failure admission, and history of coronary artery disease

^b^Model 2 was adjusted for covariates in Model 1 and laboratory findings (serum hemoglobin, albumin, creatinine, low-density lipoprotein cholesterol, corrected calcium, phosphorus, log-intact parathyroid hormone, and log-C-reactive protein levels) and temporary catheter use at hemodialysis initiation

## Discussion

In this multicenter cohort study, we found differences in patient characteristics at dialysis initiation and in the subsequent cardiac event risks following the hemodialysis initiation season. Specifically, patients who initiated hemodialysis in autumn showed volume control issues, including fluid overload and heart failure symptoms, like those observed in winter. This finding extends previous findings that fluid overload and emergency dialysis initiation due to fluid overload are most common in winter [2, 5]. To our knowledge, this is the largest study describing the patients’ features among hemodialysis initiation seasons and the first study highlighting the importance of vigilant volume control in autumn for patients with advanced chronic kidney disease to potentially reduce subsequent cardiac events by suppressing the progression of some cardiac risk factors, including LVH.

Seasonal differences in hemodialysis initiation are well recognized, but the prognostic effect of season remains unclear. To our knowledge, this relationship was examined in only one observational study using Japan’s National Medical Billing Database (NDB) [6]. They estimated the 3-month mortality rates after hemodialysis initiation based on billing data, as the NDB did not record deaths directly and found no seasonal differences. However, they did not distinguish between acute kidney injury and chronic kidney disease, nor did they give details on patient characteristics or causes of death. In contrast, our study is novel in its focus on long-term cardiac-specific prognoses, with more actual diagnoses and detailed patient information.

Cardiac risk factors at the time of hemodialysis initiation partially accounted for the differences in cardiac outcomes across the groups. For instance, the Autumn group had a higher proportion of cardiac risks (male sex, DM, higher LDL-C and CRP levels), while the Summer group had the least. This disparity was reflected in a reduction in the subdistribution hazard ratio for cardiac events from 1.70 to 1.50 following adjustment. However, even after adjustment, the Autumn group remained significantly associated with higher cardiac risk, suggesting the influence of unmeasured confounders such as left ventricular diastolic dysfunction and inflammatory markers beyond CRP. It is possible that the Autumn group included more patients with latent cardiac conditions. Supporting this, the subgroup analyses demonstrated the poorest cardiac prognosis in the Autumn group among high-risk patients (male sex, DM) and those without prior cardiac disease (heart failure, coronary artery disease).

The differences in cardiac outcomes observed in our study might be related to seasonal variations in the underlying mechanisms of kidney function decline prior to hemodialysis initiation. In summer, kidney function deterioration is frequent due to dehydration and reduced renal perfusion, particularly among patients using medications such as renin-angiotensin system inhibitors [22]. Notably, patients in the Summer group had the second highest use of renin-angiotensin system inhibitors, likely reflecting the highest prevalence of chronic glomerulonephritis. Dehydration may have caused less cardiac damage compared to fluid overload, and renin-angiotensin system inhibitors potentially contributed to improving cardiac outcome.

In contrast, winter is considered a high-risk season for kidney function deterioration due to increased salt intake, decreased physical activity, and an elevation in blood pressure resulting from cold-induced sympathetic activation. These factors not only exacerbate kidney dysfunction but also elevate cardiac risk, which may explain the poorer cardiac prognosis of patients in the Winter group compared to those in the Summer group. Additionally, fluid overload at the time of initiation may have caused greater cardiac damage compared to dehydration-induced initiation. Patients in the Spring group were likely to include those who experienced winter-related kidney function decline, as the preparation and decision-making process for dialysis often spans several weeks to months.

Autumn is a transitional period between summer and winter, exposing individuals to fluctuating temperatures. This leads to mild cold exposure without cold acclimatization, which may trigger cutaneous vasoconstriction mediated by sympathetic nerve activity [23]. Despite exhibiting high cardiac risk profiles, patients in the Autumn group had a relatively low usage of renin–angiotensin system inhibitors and diuretics. We hypothesize that the Autumn group included patients who had discontinued renin-angiotensin system inhibitors and diuretics due to summer-related kidney function decline and had not yet resumed them by autumn initiation. This led to enhanced vulnerability to mild cold stress that would typically be well tolerated, as well as increased pre-existing susceptibility to cardiac disease due to discontinuation of cardioprotective medications. Patients in the Autumn group, with vulnerability to both summer dehydration stress and cold-induced hemodynamic changes, were likely to have more severe atherosclerosis and chronic inflammation compared to the other patients. Supporting this hypothesis, the Autumn group showed the highest DM prevalence and LDL-C levels, with CRP levels comparable to those in Winter, despite the typical winter peak seasonal oscillation [24].

This study has some limitations. First, we could not investigate echocardiographic measurements thoroughly due to the limited data available from the AICOPP study. Second, this study was subjected to selection bias, because among the participants in AICOPP, chronic glomerulonephritis was less common, renal sclerosis was more frequent as an underlying disease, and the rate of patients selecting peritoneal dialysis was higher in AICOPP participants than in the nationwide data [20]. Third, survival bias was incorporated by excluding the patients who died during hospitalization for hemodialysis initiation. This exclusion caused a reduction in the apparent number of non-cardiac deaths (deaths from causes other than subsequent cardiac events). Patients who died during hospitalization may have had more severe comorbidities like acute cardiovascular disease and septic shock. Cardiovascular disease and infection increase in winter. Thus, patients in the Winter group would be more likely affected by bias than those in the other groups. Nevertheless, the exclusion would be appropriate to evaluate the risk of developing cardiac disease during the lifetime of patients on maintenance hemodialysis.

## Conclusion

Autumn initiation of hemodialysis is a possible indicator of subsequent cardiac disease development. We suggest that active coronary artery evaluation might be more beneficial for patients starting hemodialysis in autumn than in other seasons. Additionally, we suggest that more rigorous cardiac disease prevention strategies, including blood pressure control, fluid management, glycemic control, and cardioprotective medications, could be helpful for this population. A larger cohort study with comprehensive cardiac investigations and pathophysiological assessment is needed to evaluate the indicator’s reliability and utility. This research will also help improve our understanding of the mechanisms underlying cardiac diseases and their prevention.
